# Professional awareness is needed to distinguish between child physical abuse from other disorders that can mimic signs of abuse (Skull base sclerosis in infant manifesting features of infantile cortical hyperostosis): a case report and review of the literature

**DOI:** 10.1186/1757-1626-2-133

**Published:** 2009-02-09

**Authors:** Ali Al Kaissi, Gert Petje, Veerla De Brauwer, Franz Grill, Klaus Klaushofer

**Affiliations:** 1 Ludwig Boltzmann Institute of Osteology, at the Hanusch Hospital of WGKK and, AUVA, Trauma Centre Meidling, 4th Medical Department, Hanusch Hospital, Vienna, Austria; 2 Orthopaedic Hospital of Speising, Paediatric Department, Vienna, Austria

## Abstract

**Background:**

Infantile cortical hyperostosis is characterised by hyperirritability, acute inflammation of soft tissue, and profound alterations of the shape and structure of the underlying bones, particularly the long bones, mandible, clavicles, or ribs.

**Case presentation:**

We report on a clinical case of a 3-months-old baby girl of non-consanguineous parents. Multiple long bone swellings were the motive of referral to our department for clinical evaluation. Radiographic documentation was consistent with infantile cortical hyperostosis (Caffey disease). Interestingly, skull base sclerosis associated with excessive thickening was the most unusual malformation. We report a baby with mixed endochondral and intramembraneous ossification defects.

**Conclusion:**

Bone dysplasias, mucopolysaccharidoses, and metabolic diseases are a group of disorders that cause abnormal growth, density, and skull base shape. Skull base sclerosis/thickening is a well-known malformation in connection with other forms of sclerosing bone disorders such as dysosteosclerosis, frontometaphyseal dysplasia, and progressive diaphyseal dysplasia with skull base involvement. It is noteworthy that our present patient had an unusually sclerosed/thickened skull base. Narrowing of skull foramina due to sclerosis of skull base is likely to result in cranial nerves deficits. In this baby, the pathology has been judged to be the result of child abuse and it is not, in this case considerable harm to his parents, and the doctor-parent relationship was the outcome.

## Background

Infantile cortical hyperostosis is a genetic disorder described by Caffey and Silverman. It is characterised by an infantile episode of massive subperiosteal new bone formation that typically involve the diaphyses of the long bones, mandible and clavicle [1,2]. The involved bones may appear inflamed, with painful swelling and systemic fever often accompanying the illness. Autosomal dominant inheritance was documented on several occasions [3-5] but expressivity is variable and non-penetrance exists. Many hypotheses for its pathogenesis have been suggested such as infection, genetic, immunological, allergic and vascular origins, but no cause has been proven to date. The radiological differential diagnosis should include osteomyelitis, vitamin A intoxication, scurvy, syphilis, osteosarcoma and battered child syndrome. The radiographic features of infantile cortical hyperostosis evolve in stages. Hyperostosis develops on the outer cortical surface, expand, and then remodel by resorption either at the external surface or at the endocortical surface. In the latter case, there is expansion of the medulla and the cortex is thin. Tubular bones may be grossly deformed, with bony bridges sometimes appearing between adjacent bones (as seen in the radius of our patient). Autosomal dominant trait with incomplete penetrance has been postulated [6-9].

## Case presentation

A 3-months-old female was referred to our department because of suspicion of child physical abuse. She was born at term, a product of uneventful gestation. At birth her weight was 3310 gm, length of 51 cm and her occipito-frontal circumference was 33.5 cm. No irritability, feeding or pulmonary abnormalities, and fever were recorded. The mother was 29-year-old-gravida 2, married to a 33-year-old-unrelated man. She had no history of spontaneous abortions, stillbirths, prematurity and or polyhydramnios. No maternal use of medications or antenatal illnesses was reported. Physical examination of the baby showed normal facial features, no blue sclerae, normal nose but swelling of the mandible was noted. There were swellings of some of the long bones (right leg, and radius). Neither feeding abnormalities nor fever was present. Her chest was noted to be symmetrical and of normal appearance. Hands and feet were normal. There were no associated signs of abnormal skin stigmata such as fragility/extensibility and or ligamentous hyperlaxity was noted. Hearing, vision and neurological examinations were all normal. Laboratory studies including metabolic tests, which aimed to tests calcium, phosphorus, and vitamin D metabolism were normal. No specific genetic test has been done for this baby.

**Figure 1:**
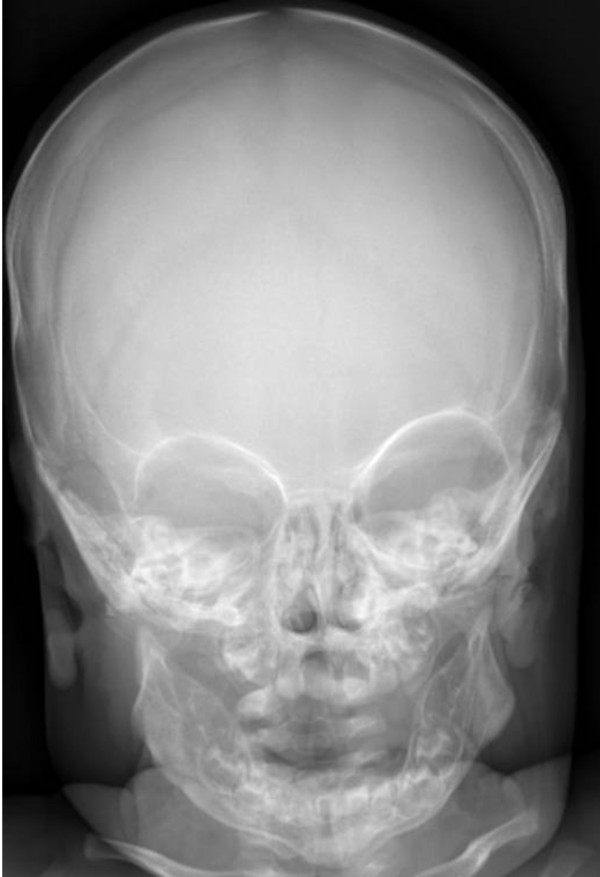
**Anteroposterior radiograph of the skull showed massive sclerosis of the skull bone associated with significant cortical hyperostosis and enlargement of the mandible secondary to cortical new bone formation**.

**Figure 2:**
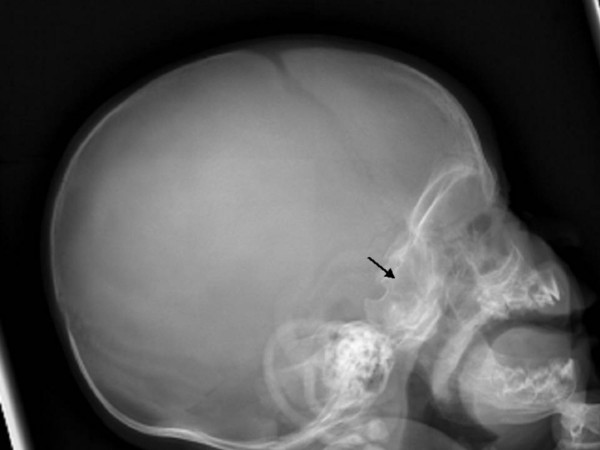
**Lateral skull radiograph showed sclerosis of the skull base and hyperostosis of the calvaria (fig 2)**.

**Figure 3:**
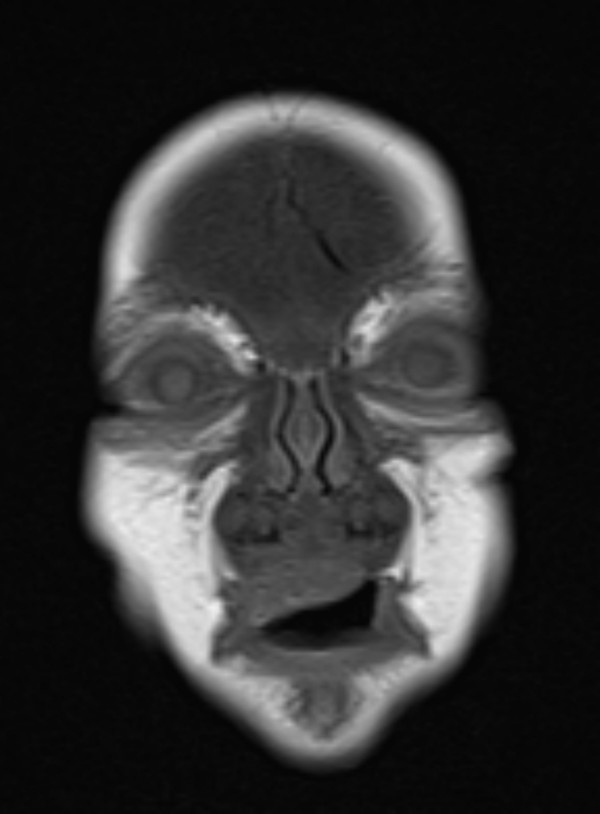
**Coronal MRI imaging showed significant calvarial/facial and mandibular hyperostosis**.

**Figure 4:**
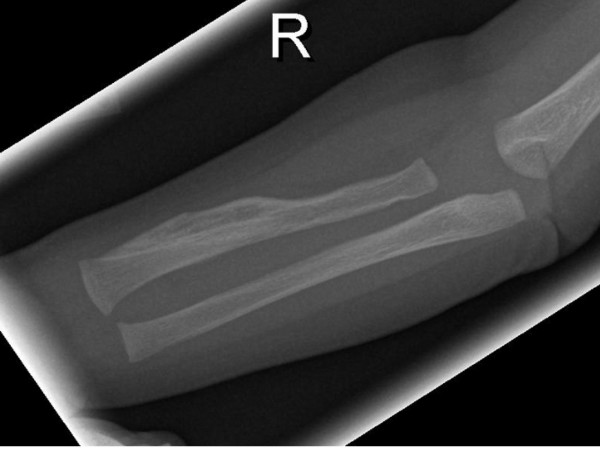
**Anteroposterior radiograph of the radius showed cortical new bone formation associated with subperiosteal thickening**. Note marked bloating along the diaphysis with sparing of the epi-metaphyseal components associated with expansion of the bone marrow cavity and a persistent-like deformity (fig 4).

**Figure 5:**
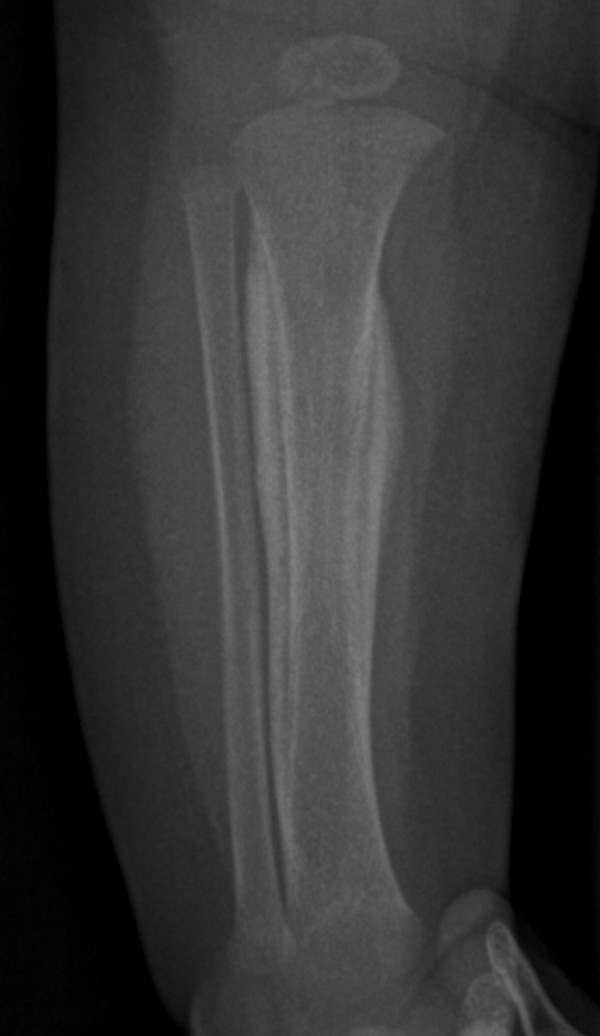
**Anteroposterior radiograph of the tibia showed a thick and broad ballooning occupies the diaphyses (proximally and distally) there is gaining in the diameter comes from subperiosteal new bone apposition by intramembraneous bone formation**.

Anteroposterior radiograph of the skull showed massive sclerosis of the skull bone associated with significant cortical hyperostosis and enlargement of the mandible secondary to cortical new bone formation (fig 1). Lateral skull radiograph showed sclerosis of the skull base (arrow) and hyperostosis of the calvaria (fig 2). Coronal MRI imaging showed significant calvarial/facial and mandibular hyperostosis (fig 3). Anteroposterior radiograph of the radius showed cortical new bone formation associated with subperiosteal thickening. Note marked bloating along the diaphysis with sparing of the epi-metaphyseal components associated with expansion of the bone marrow cavity and a persistent-like deformity (fig 4). Anteroposterior radiograph of the tibia showed a thick and broad ballooning occupies the diaphyses (proximally and distally) there is gaining in the diameter comes from subperiosteal new bone apposition by intramembraneous bone formation (fig 5).

## Discussion

Skull base is a site for endochondral ossification. Typical target sites for endochondral ossification where the process takes place are the tubular and flat bones, vertebrae, skull base, ethmoids, and the end of the clavicle. On the other hand, direct transformation of condensed mesenchymal cells, without an intervening cartilage stage, results in the formation of cortical bone. This bony apposition starts with formation of a periosteal collarbone at the ninth week of fetal life. Target sites for intramembranous ossification include the cortex of tubular and flat bones, the calvaria, the bones of the upper face, and tympanic parts of the temporal bone, vomer and medial pterygoid [10,11]. In mixed sclerosing dysplasias, abnormal sclerosis is the consequence of disturbances in both endochondral and intramembreneous ossification. In dysosteosclerosis, metaphyseal dysplasia (Pyle's disease), frontometaphyseal dysplasia, endochondral ossification defects predominate. Intramembraneous ossification defects might be encountered in a number of skeletal dysplasias such as melorheostosis, craniodiaphyseal dysplasia, Lenz-Majewski hyperostotic dwarfism, and progressive diaphyseal dysplasia with skull base involvements [12].

Infantile cortical hyperostosis is an inherited disorder characterised by hyperirritability, acute inflammation of soft tissues, and massive subperiosteal new bone formation. There is onset of pain and swelling before the age of 6 months, often in the lower limbs or jaws, and sometimes accompanied by fever. Both the clinical and radiological features usually disappear before the age of 1 year. The mandible, ribs, clavicles and cranium can all be affected. The pattern of distribution of the lesions varies from patient to patient and can be symmetrical. Swellings also involve the underlying muscles. The phase of acute inflammation precedes the abnormal thickening of the cortical bone (hyperostosis). It also subsides long before the hyperostosis resolves. Sometimes lesions recur suddenly in their original sites or new sites, either during or after the subsidence of the swellings that appeared at the onset of the disease. This uneven protracted clinical course with unpredictable remissions and relapses as one of the characteristic features of this disorder. Bull and Feingold [13] reported 2 affected sisters, one of whom had affected son and daughter and the other a normal daughter and affected son. Fried et al. [14] observed 9 affected persons in 3 sibships of 2 generations of a family. It has been suggested, that infantile cortical hyperostosis is closely associated with a mutation in COL1A1, the gene encoding the alpha chain of type I collagen. Of the 24 affected members of a family segregating Caffey disease in which Gensure et al [9] identified an R836C mutation in the COL1A1 gene, only 19 (79%) had experienced an episode of cortical hyperostosis and 5 (21%) obligate carriers had not, consistent with reduced penetrance. Skull base sclerosis/thickening was not a feature in the above-mentioned reports.

## Conclusion

Multiple inflamed swellings over the limbs associated with radiographic features of callus-like were the reasons to suspect the current baby as physically abused. Inconsistent explanation for swellings, fractures, head trauma, or bruises in a child is among the red flags that raise suspicion of physical abuse. Child abuse remains one of the most misdiagnosed problems in all of paediatrics. On one hand, a lack of awareness of their own social biases, coupled with a lack of knowledge about disorders that can mimic signs of abuse, can mislead doctors to see child abuse where there is none and vice versa. Finally we wish to stress that in children with unexplained traumas/fractures, it is empirical to review carefully their clinical history, family history, physical examinations of the parents and proper radiographic interpretation.

## Abbreviations

ICH: Infantile cortical hyperostosis

## Competing interests

The authors declare that they have no competing interests.

## Authors' contributions

All of the authors were involved in the clinico-radiographic assessment and finalising the paper. All authors have red and approved the final version of the paper.

## Consent

Written informed consent was obtained from the parents for the purpose of publication of the manuscript and figures of their child. A copy of the written consent is available for review by the editor-in-Chief of this journal.

## References

[B1] Caffey J Infantile cortical hyperostosis: a review of the clinical and radiographic features Proc R Soc Med 1957 50 347 354 13431894 10.1177/003591575705000516 PMC1889299

[B2] Caffey J Silverman WA Infantile cortical hyperostoses, preliminary report on a new syndrome Am J Roentgenol 1945 54 1 16

[B3] Borochowitz Z Gozal D Misselevitch I Familial Caffey's disease and late recurrence in a child Clin Genet 1991 40 329 335 1756606 10.1111/j.1399-0004.1991.tb03104.x

[B4] Clemett AR Williams JH The familial occurrence of infantile cortical hyperostosis Radiology 1963 80 409 416 14021697 10.1148/80.3.409

[B5] Bull MJ Feingold M Autosomal dominant inheritance of Caffey disease BDOAS 1974 10 9 139 146 4609117

[B6] Kuhl J Harris GB Maroteaux P Ein Beitrag zur Krankheitsbild der infantilen kortikalen Hyperostose Arch Kinderheilkd 1969 179 209 229 4900471

[B7] Langer R Kaufmann HJ Prenatal diagnosis of Caffey's disease (infantile cortical hyperostosis) (in German – summary in English) Klin Padiatr 1985 197 473 476 10.1055/s-2008-1034024 3910953

[B8] Taj-Eldin S Al-Jawad J Cortical hyperostosis. Infantile and juvenile manifestations in a boy Arch Dis Child 1971 46 565 566 4935623 10.1136/adc.46.248.565 PMC1647770

[B9] Gensure RC Makitie O Barclay C Chan C DePalma SR Bastepe M Abuzahra H Couper R Mundlos S Sillence D Ala Kokko L Seidman JG Cole WG Juppner H A novel COL1A1 mutation in infantile cortical hyperostosis (Caffey disease) expands the spectrum of collagen-related disorders J Clin Invest 2005 115 1250 1257 15864348 10.1172/JCI22760 PMC1087158

[B10] Greenspan A Sclerosing bone dysplasias: a target-site approach Skeletal Radiol 1991 20 561 583 10.1007/BF01106087 1776023

[B11] Soames RW Williams PL Histogenesis of bone Gray's anatomy 1995 Churchill Livingstone, London 471 478

[B12] Maroteuax P Le Merrer M Maladies osseuses de l' enfant 2002 4 Paris: Medeicine-Sciences Flammarion

[B13] Bull MJ Feingold M Autosomal dominant inheritance of Caffey disease BDOAS 1974 10 9 139 146 4609117

[B14] Fried K Manor A Pajewski M Autosomal dominant inheritance with incomplete penetrance of Caffey disease (infantile cortical hyperostosis) Clin Genet 1981 19 271 274 7023758 10.1111/j.1399-0004.1981.tb00708.x

